# Case report: Gait-induced palilalia in a patient with hemiplegia due to cerebral infarction

**DOI:** 10.3389/fnhum.2024.1361585

**Published:** 2024-07-03

**Authors:** Yoshiyuki Kaneko, Takahiro Suzuki, Hideto Nakajima, Tadashi Kanamori, Masahiro Suzuki

**Affiliations:** ^1^Department of Psychiatry, Nihon University School of Medicine, Tokyo, Japan; ^2^Division of Neurology, Department of Internal Medicine, Nihon University School of Medicine, Tokyo, Japan

**Keywords:** palilalia, cerebral infarction, hemiplegia, network reorganization, carbamazepine

## Abstract

**Background:**

Palilalia is a type of speech characterized by compulsive repetition of words, phrases, or syllables. Several reports have noted that palilalia can occur in response to external verbal stimuli. Here, we report, for the first time, a patient with palilalia induced by gait, which we call “movement-related palilalia.”

**Case presentation:**

Eleven months after the onset of cerebral infarction sparing the right precentral gyrus and its adjacent subcortical regions, a 63-year-old, left-handed Japanese man was referred for psychiatric consultation because of a complaint of irritability caused by the stress of compulsive repetition of a single meaningless word, “wai.” The repetition of a word, palilalia, in this case, was characterized by its predominant occurrence during walking and by its melodic tones. The palilalia during walking disappeared almost completely after 5 months of treatment with carbamazepine 600 mg.

**Conclusion:**

Palilalia induced by gait can occur in patients with a history of cerebral infarction. This palilalia during walking may be due to the reorganization of networks in areas nearby or surrounding cerebral infarcts.

## 1 Introduction

Palilalia is a type of reiterative speech characterized by the involuntary repetition of syllables, words, phrases, or sentences (Critchley, 1927). This phenomenon has been observed in patients with various neurological disorders (Stracciari et al., 1993; Benke and Butterworth, 2001; Landi et al., 2012; Patira et al., 2017). Here, we report the extremely rare case of a patient with hemiplegia due to cerebral infarction who experienced palilalia during walking. Palilalia in this case was induced when the patient attempted to move his paralyzed left lower limb.

## 2 Case description

**Figure 1:**
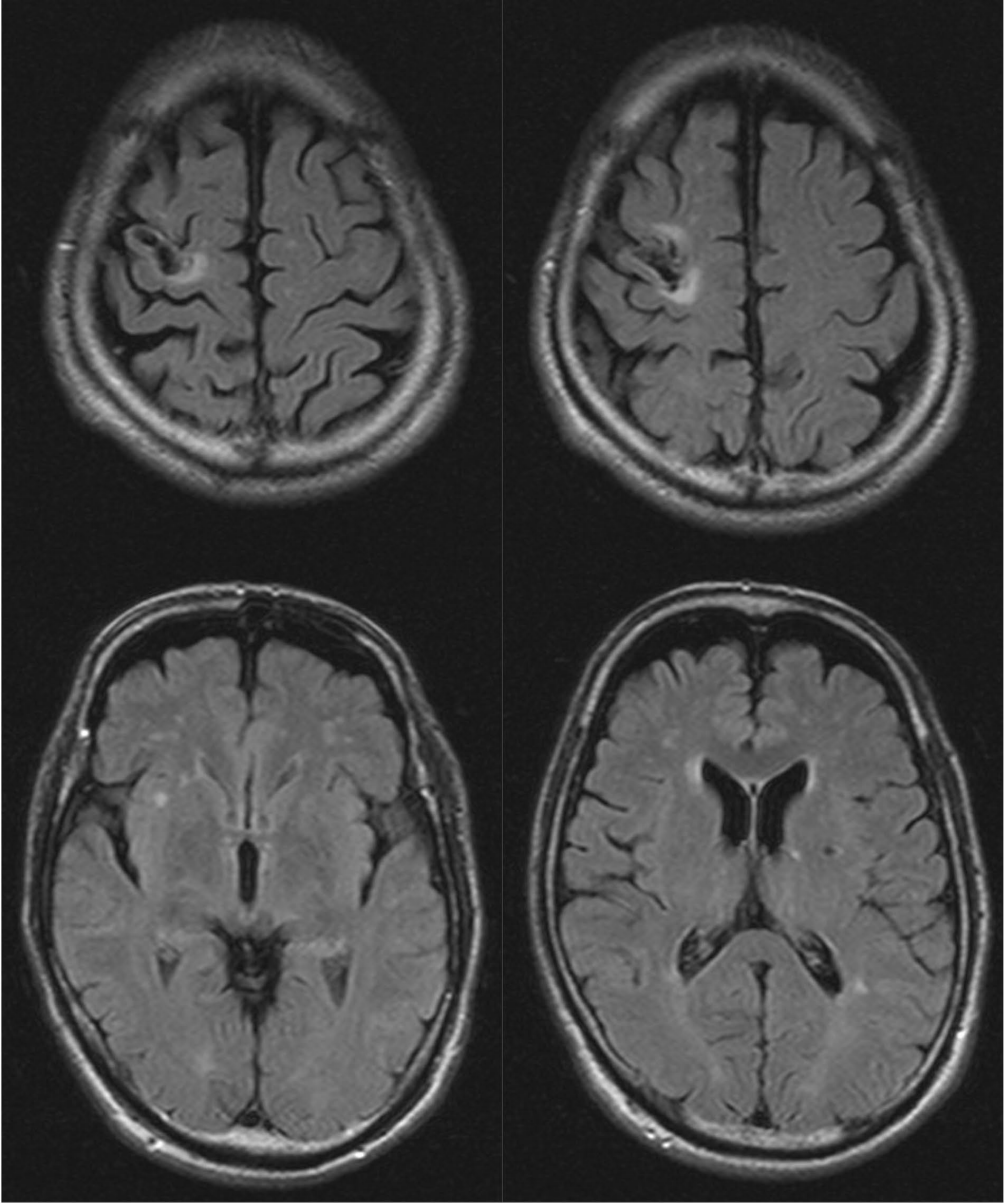
Fluid-attenuated inversion recovery (FLAIR) images of the patient’s brain.

A 63-year-old, left-handed, Japanese man was admitted to the neurosurgery unit of a general hospital with a sudden onset of left hemiplegia and was diagnosed with infarction of the right frontoparietal lobe. He received conservative treatment, but his hemiplegia remained. Eight months after the stroke, he lost consciousness transiently and fell. At that time, the patient was diagnosed with suspected post-stroke epilepsy at a neurosurgery clinic and treated with levetiracetam. Three months later, he started to notice the involuntary repetition of a meaningless syllable, “wai.” The antiepileptic regimen was changed to lacosamide; however, the involuntary utterances did not decrease. The patient was then referred for psychiatric consultation because of impulsive aggression after the cerebral infarction. These consultations showed that repeated involuntary utterances, identified as palilalia, had two unique characteristics: They occurred predominantly while walking and were delivered in melodic tones. Detailed observation showed that the palilalia appeared a few seconds before he started to walk and that the rate of palilalia was synchronized with walking (Supplementary Video 1). The patient was unable to stop or limit the palilalia by any means other than stopping walking. Even after stopping walking, the palilalia often continued for a few minutes, but it gradually lost its melodic nature. Palilalia with a melodic pattern was predominantly induced when he tried to bend and stretch his left knee (Supplementary Video 2). The patient had a history of diabetes mellitus, dyslipidemia, and abdominal aortic aneurysm but no significant family history. Neurological examination by an expert (HN) revealed left-sided spastic hemiparesis and gait festination. The Mini-Mental State Examination score was 15 (maximum, 30), with points lost for orientation to time and place, calculation, recall, language, and construct. The aphasia quotient from the Western Aphasia Battery score was 39, suggesting severe aphasia. The patient displayed difficulty with spontaneous speech, and his speech was non-fluent. He also showed difficulty repeating long sentences, mainly because the palilalia interrupted him. He did not exhibit any impairment of word recall or prosody, but he had some difficulty comprehending long or complex sentences. Magnetic resonance imaging (MRI) showed infarction sparing the right precentral gyrus and the adjacent subcortical region, as well as small infarcts in the bilateral basal ganglia and chronic ischemic changes in the white matter (Figure 1). Video electroencephalogram monitoring showed no epileptiform discharges, even when palilalia was induced by bending and stretching the left knee. Pharmacotherapy with carbamazepine 200 mg/day was started for the aggression, and the palilalia gradually decreased after the dose was increased to 400 mg/day for a month. Palilalia during walking disappeared after 5 months of treatment with carbamazepine 600 mg/day (Supplementary Video 3). The patient adhered well to the treatment and experienced no significant side effects. His aggression decreased as the palilalia during walking disappeared. No obvious epileptic manifestations were observed while taking antiepileptic drugs. The patient provided written informed consent for the publication of any potentially identifiable images or data included in this article.

## 3 Discussion

Palilalia, as a compulsive repetition of words, phrases, or syllables, has been described in several neurological disorders, including cerebrovascular (Benke and Butterworth, 2001) and degenerative diseases (Stracciari et al., 1993), encephalitis (Patira et al., 2017), and epilepsy (Landi et al., 2012). Palilalia has been observed in spontaneous speech (Gorno et al., 1997; Patira et al., 2017), in response to a question (Dierckx et al., 1991; Ueki et al., 2000), and in both contexts (Critchley, 1927; Van Borsel et al., 2007). Palilalia, in the present case, was characterized by being induced by limb movements. To the best of our knowledge, this is the first report to describe palilalia induced by body movements. We have called this phenomenon “movement-related palilalia.” In the present case, a specific melodic pattern was uttered when the patient with a history of cerebral infarction intended to move his paralyzed limb, suggesting that attempts to move the paralyzed limb were activating neuronal networks corresponding to the specific melodic pattern. The right premotor cortex, which had been damaged in the present case, is an area associated with not only motor preparation but also melody generation (De Manzano and Ullen, 2012). Generally, brain functions are known to undergo reorganization in areas close to or surrounding cerebral infarcts several months after stroke onset (Cramer et al., 1997; Traversa et al., 1997). Movement-related palilalia in the present case appeared to be attributable to the reorganization of the networks related to limb movements and melody generation, based on the characteristics of palilalia appearing several months after the onset of cerebral infarction and the fact that the symptoms were relieved by carbamazepine. Carbamazepine is a drug that is effective against post-stroke epilepsy (Tanaka and Ihara, 2017) and phantom limb pain (Patterson, 1988), which have similarly been considered to result from cortical reorganization (Maclver et al., 2008; Tanaka and Ihara, 2017). In light of such findings from previous studies, carbamazepine in the present case may have played a role in regulating the reorganized network. Lacosamide was also used in the present case but had no effect. Carbamazepine and lacosamide inhibit voltage-gated sodium channels; however, their pharmacological properties differ. Lacosamide facilitates slow inactivation of voltage-gated sodium channels, whereas carbamazepine inactivates voltage-gated sodium channels much more rapidly. The slow inactivation induced by lacosamide is thought to be relatively selective for repeatedly depolarizing neurons, such as those participating in seizure activity (Curia et al., 2009). Therefore, carbamazepine may be more effective than lacosamide in a non-epileptic condition such as the present case. Further investigations with more cases are needed to identify the pathophysiology underlying this phenomenon and to develop treatment methods.

We have discussed the possibility that the symptoms in the present case could be due to a disturbance in the right premotor cortex, but the lesion responsible for palilalia in the present case is debatable. In addition to the right premotor cortex, small lesions were also observed in the basal ganglia bilaterally, a region involved in motor programming. Since the basal ganglia are also considered to be involved in palilalia (Critchley, 1927; Ikeda and Tanabe, 1992), it is possible that this region was involved in the development of symptoms in the present case. The possibility that the damage in these regions was involved in the development of the symptoms in the present case cannot be ruled out. Functional brain imaging tests such as functional MRI or neurophysiological techniques, including event-related potentials, may be useful for identifying the lesion responsible for palilalia in the present case.

In conclusion, we reported a case of a patient with palilalia that occurred 11 months after cerebral infarction, sparing the right precentral gyrus and adjacent subcortical regions. The palilalia in the present case was in melodic tones and was induced by gait. The present case suggests that cerebral infarction in areas including the right premotor cortex may cause palilalia during walking via reorganization of networks in areas close to or surrounding cerebral infarcts.

## Data availability statement

The datasets presented in this article are not readily available because of ethical and privacy restrictions. Requests to access the datasets should be directed to the corresponding author.

## Ethics statement

Ethical review and approval was not required for the study on human participants in accordance with the local legislation and institutional requirements. The studies were conducted in accordance with the local legislation and institutional requirements. The participants provided their written informed consent to participate in this study. Written informed consent was obtained from the individual for the publication of any potentially identifiable images or data included in this article.

## Author contributions

YK: Conceptualization, Investigation, Methodology, Project administration, Visualization, Writing – original draft. TS: Investigation, Writing – review & editing. HN: Investigation, Writing – review & editing. TK: Investigation, Writing – review & editing. MS: Project administration, Supervision, Writing – review & editing.

## References

[ref1] Benke T. Butterworth B. (2001). Palilalia and repetitive speech: two case studies. Brain Lang. 78, 62–81. doi: 10.1006/brln.2000.2445, PMID: 11412016

[ref2] Cramer S. C. Nelles G. Benson R. R. Kaplan J. D. Parker R. A. Kwong K. K.. (1997). A functional MRI study of subjects recovered from hemiparetic stroke. Stroke 28, 2518–2527. doi: 10.1161/01.STR.28.12.2518, PMID: 9412643

[ref3] Critchley M. (1927). On palilalia. J. Neurol. Psychopathol. 8, 23–32. doi: 10.1136/jnnp.s1-8.29.23, PMID: 21611242 PMC1068500

[ref4] Curia G. Biagini G. Perucca E. Avoli M. (2009). Lacosamide: a new approach to target voltage-gated sodium currents in epileptic disorders. CNS Drugs 23, 555–568. doi: 10.2165/00023210-200923070-00002, PMID: 19552484 PMC4878900

[ref5] De Manzano O. Ullen F. (2012). Activation and connectivity patterns of the presupplementary and dorsal premotor areas during free improvisation of melodies and rhythms. NeuroImage 63, 272–280. doi: 10.1016/j.neuroimage.2012.06.024, PMID: 22732560

[ref6] Dierckx R. A. Saerens J. De Deyn P. P. Verslegers W. Marien P. Vandevivere J. (1991). Evolution of technetium-99m-HMPAO SPECT and brain mapping in a patient presenting with echolalia and palilalia. J. Nucl. Med. 32, 1619–1621, PMID: 1869990

[ref7] Gorno M. L. Miozzo A. Mattioli F. Cappa S. F. (1997). Isolated palilalia: a case report. Eur. J. Neurol. 4, 94–96. doi: 10.1111/j.1468-1331.1997.tb00306.x, PMID: 24283829

[ref8] Ikeda M. Tanabe H. (1992). Two forms of palilalia: a clinicoanatomical study. Behav. Neurol. 5, 241–246. doi: 10.1155/1992/824182, PMID: 24487810

[ref9] Landi D. Benvenga A. Quattrocchi C. C. Vollero L. Assenza G. Pellegrino G.. (2012). Complex epileptic palilalia: a case report. Seizure 21, 655–657. doi: 10.1016/j.seizure.2012.06.009, PMID: 22776676

[ref10] Maclver K. Lloyd D. M. Kelly S. Roberts N. Nurmikko T. (2008). Phantom limb pain, cortical reorganization and the therapeutic effect of mental imagery. Brain 131, 2181–2191. doi: 10.1093/brain/awn124, PMID: 18567624 PMC2494616

[ref11] Patira R. Smith-Benjamin S. Ramachandran V. S. Altschuler E. L. (2017). Palilalia due to steroid-responsive encephalopathy. Neurol Clin Pract. 7, e23–e25. doi: 10.1212/CPJ.0000000000000278, PMID: 30107006 PMC6081963

[ref12] Patterson J. F. (1988). Carbamazepine in the treatment of phantom limb pain. South. Med. 81, 1100–1102. doi: 10.1097/00007611-198809000-00008 3047877

[ref13] Stracciari A. Guarino M. Cirignotta F. Pazzaglia P. (1993). Development of palilalia after stereotaxic thalamotomy in Parkinson's disease. Eur. Neurol. 33, 275–276. doi: 10.1159/000116953, PMID: 8467853

[ref14] Tanaka T. Ihara M. (2017). Post-stroke epilepsy. Neurochem. Int. 107, 219–228. doi: 10.1016/j.neuint.2017.02.002 28202284

[ref15] Traversa R. Cicinelli P. Bassi A. Rossini P. M. Bernardi G. (1997). Mapping of motor cortical reorganization after stroke. A brain stimulation study with focal magnetic pulses. Stroke 28, 110–117. doi: 10.1161/01.STR.28.1.110 8996498

[ref16] Ueki Y. Kohara N. Oga T. Fukuyama H. Akiguchi I. Kimura J.. (2000). Membranous lipodystrophy presenting with palilalia: a PET study of cerebral glucose metabolism. Acta Neurol. Scand. 102, 60–64. doi: 10.1034/j.1600-0404.2000.102001060.x, PMID: 10893065

[ref17] Van Borsel J. Bontinck C. Coryn M. Paemeleire F. Vandemaele P. (2007). Acoustic features of palilalia: a case study. Brain Lang. 101, 90–96. doi: 10.1016/j.bandl.2006.06.118, PMID: 16890278

